# Spatial analysis and characteristics of pig farming in Thailand

**DOI:** 10.1186/s12917-016-0849-7

**Published:** 2016-10-06

**Authors:** Weerapong Thanapongtharm, Catherine Linard, Pornpiroon Chinson, Suwicha Kasemsuwan, Marjolein Visser, Andrea E. Gaughan, Michael Epprech, Timothy P. Robinson, Marius Gilbert

**Affiliations:** 1 Department of Livestock Development (DLD), Bangkok, 10400 Thailand; 2 Lutte biologique et Ecologie spatiale (LUBIES), Université Libre de Bruxelles, Brussels, 1050 Belgium; 3 Fonds National de la Recherche Scientifique (FNRS), Brussels, 1050 Belgium; 4 Faculty of Veterinary Medicine, Kasetsart University, Kampangsaen Campus, Nakornpatom, 73140 Thailand; 5 Research Unit of Landscape Ecology AND Plant Production Systems (EPSPV), University of Brussels, 1050 Brussels, Belgium; 6 Department of Geography and Geosciences, University of Louisville, Louisville, 40292 USA; 7 Centre for Development and Environment (CDE), Country office in the Lao PDR, Vientiane, 6101 Lao PDR; 8 Livestock Systems and Environment (LSE), International Livestock Research Institute (ILRI), Nairobi, 30709 Kenya

**Keywords:** Intensive pig farm, Sustainable development, Spatial distribution, Random forest, Two-part model

## Abstract

**Background:**

In Thailand, pig production intensified significantly during the last decade, with many economic, epidemiological and environmental implications. Strategies toward more sustainable future developments are currently investigated, and these could be informed by a detailed assessment of the main trends in the pig sector, and on how different production systems are geographically distributed. This study had two main objectives. First, we aimed to describe the main trends and geographic patterns of pig production systems in Thailand in terms of pig type (native, breeding, and fattening pigs), farm scales (smallholder and large-scale farming systems) and type of farming systems (farrow-to-finish, nursery, and finishing systems) based on a very detailed 2010 census. Second, we aimed to study the statistical spatial association between these different types of pig farming distribution and a set of spatial variables describing access to feed and markets.

**Results:**

Over the last decades, pig population gradually increased, with a continuously increasing number of pigs per holder, suggesting a continuing intensification of the sector. The different pig-production systems showed very contrasted geographical distributions. The spatial distribution of large-scale pig farms corresponds with that of commercial pig breeds, and spatial analysis conducted using Random Forest distribution models indicated that these were concentrated in lowland urban or peri-urban areas, close to means of transportation, facilitating supply to major markets such as provincial capitals and the Bangkok Metropolitan region. Conversely the smallholders were distributed throughout the country, with higher densities located in highland, remote, and rural areas, where they supply local rural markets. A limitation of the study was that pig farming systems were defined from the number of animals per farm, resulting in their possible misclassification, but this should have a limited impact on the main patterns revealed by the analysis.

**Conclusions:**

The very contrasted distribution of different pig production systems present opportunities for future regionalization of pig production. More specifically, the detailed geographical analysis of the different production systems will be used to spatially-inform planning decisions for pig farming accounting for the specific health, environment and economical implications of the different pig production systems.

**Electronic supplementary material:**

The online version of this article (doi:10.1186/s12917-016-0849-7) contains supplementary material, which is available to authorized users.

## Background

In the recent decades, changes in the pig production sector have occurred in many countries, enabling increases in production of pig meat per capita and per farm [1, 2]. The changes to the production systems included a shift from extensive, small-scale, subsistence, mixed production systems towards more intensive, large-scale, geographically-concentrated, commercially-oriented and specialized production [1]. In Thailand, this process of intensification started in the 1960s when the first commercial pig breeds were imported from the United Kingdom by the Department of Livestock Development (DLD) and then from the United States by Kasetsart University [2]. Since then, smallholders who raise indigenous native pig breeds for both personal consumption and as a supplementary source of income have been gradually replaced by large-scale farming of improved pig breeds [4, 5]. The pig revolution in Thailand corresponds to the introduction of modern technologies and farm management. The introduction of modern technology include the use of evaporated cooling animal housing, which provides temperatures ranging between 25 and 27 °C (pigs are particularly susceptible to heat stress) artificial insemination, and optimized feed ingredients and additives. These combined factors have allowed commercial farmers to raise more pigs per square meter with faster production cycles [2]. These production systems are referred to as ‘intensive’ in the sense that a high amount of infrastructure, technology, health care and feeds are used to increase the productivity of high-yielding animals on the farm, resulting in increased outputs (kg meat per animal space per year) [3]. In the pig sector, intensive production systems characterized by high input/output ratios generally, also correspond to large farm size. Although intensive systems could also be obtained in small-scale farming, using high inputs of manpower for example, this does not correspond to the current situation in the Asian region. The very large majority of smallholders use very low levels of inputs in their production cycle, have limited outputs in return, and can therefore be characterized as extensive. Consequently, in Asia, pig production systems are still largely classified in extensive vs. intensive by their farm size, expressed as number of head per farm. For example, following an extensive review of the farm-sizes in different countries, Robinson et al. used thresholds of 10 and 100 pigs/farm to distinguish extensive (<10), semi-intensive (10–100) and intensive (>100) pig farming systems [1].

There is a strong link between the occurrence of diseases, pig production systems and farm scales [3–5]. Smallholders pig production systems are usually linked to poor hygiene and low bio-security with few barriers to potential contacts between the pigs, humans and wildlife. This facilitates disease transmission from wildlife to pig, pig to pig and pig to human. A typical example of disease affecting smallholders in Thailand is trichinosis, a parasitic disease circulating in wild and domestic animals such as rats, pigs, and wild pigs, and occasionally infecting human through the consumption of inadequately cooked infected pork [6]. So, smallholders are characterized by endemic and parasitic diseases with a relatively limited impact. In contrast, intensive pig production systems are hosts to other types of diseases. The hygiene and bio-security can be much higher than in small-scale production systems, but the high concentrations of genetically similar animals, sharing a limited space and producing large quantities of effluent results in i) increased contact rates and pathogen transmission within and between these populations, ii) the build-up of potential pathogens in the environment and in carrier animals e.g. older breeding stock; and iii) the emergence of new serotypes or mutations [4, 5]. For example, an atypical and highly virulent form of Porcine Reproductive and Respiratory Syndrome (PRRS) recently emerged in pig farms in China [7] and spread to many other countries throughout Asia resulting in a significant productivity impact in the pig production systems [8–12]. Swine influenza is endemic in the pig production sector, but one of the few factors positively associated with disease risk is the farm size [13]. Intensive pig production also has an indirect potential effect through the emergence of zoonotic diseases. The concurrence of several conditions such as high densities of pigs and farms, together with the immunological characteristics of pigs themselves, increase the chance of emergence and spread of some zoonotic pathogens that originate from wild animals passing to pigs (called “mixing vessel”) and then on to humans [14]. For examples, pigs have been identified as mixing vessels for influenza viruses [15] – having receptors both for avian and mammalian viruses - and as intermediate hosts for Nipah viruses [16, 17]. In environmental terms, intensive pig production systems are also a serious cause of environmental pollution, both air and water, due to poor manure management [18]. Intensive pig production systems can also radically alter biodiversity of aquatic ecosystems because water polluted by manure that is rich in phosphates, nitrates, and organic matter stimulates the growth of oxygen-depleting plant life, such as blue algae, that then affects fisheries and other valuable aquatic biodiversity [18].

In Thailand, pig farming systems can be categorized into three groups: i) the farrow-to-finish production system, which includes breeding pigs, producing piglets and fattening pigs in the same farm; ii) the nursery system, which only raises breeding pigs to produce piglets; and iii) the finishing system, which raises weaners until they reach market weight [19, 20]. Nowadays, two groups of pig breeds are used in Thailand: the native breeds such as Raad or Ka Done, Puang, Hailum, Kwai, and wild pigs ([21, 22] and the main commercial breeds, including the Large White, Landrace, Duroc, and crosses of these [20]. Native pig breeds grow slowly and their reproduction rates are lower than those of commercial breeds. However, they are better adapted to hot and humid climates and to low-quality feed [21] and they apparently show higher resistance to endemic diseases such as Foot and Mouth Disease (FMD) and internal parasites [21]. In contrast, commercial pig breeds grow much faster, with comparatively higher feed conversion rates and their carcass and meat quality better meet supermarket needs for standardized products [2].

Previous studies demonstrated that farm-level characteristics (i.e. production systems), could be an important risk factor for different diseases in Thailand [2, 12, 23]. For examples, the movements of pigs between production stages provide significant opportunities for the transmission of diseases between herds or farms. Examples include Transmissible Gastroenteritis (TGE) and PRRS [5]. Purchasing feeder pigs from outside the farm increases the risk of introducing diseases such as PRRS, Classical Swine Fever (CSF), and FMD [2]. Farms with breeding sows are at a higher risk from PRRS [12]. The traditional farrow-to-finish system, with high levels of mixing between age groups, facilitates the exchange of a wide number of potential pathogens within the farm, especially enteric and respiratory diseases [23]. In terms of environmental impacts, the Thailand Pollution Control Department (PCD) reported that the high concentration of pig farms in the central plain caused significant water pollution in rivers, and consequently, PCD added pig farming to the list of regulated activities in 2001 [2, 24].

In order to reduce the adverse impacts of intensive pig farming, both in epidemiological and environmental terms, the Agricultural Standard Committee (Ministry of Agriculture and Cooperatives MOAC, Thailand), established the “Standard for Good Agricultural Practices for Pig Farms”, which aimed to provide guidance to pig farmers and promote healthy and hygienic pig farming practices [25]. This document provides recommendations relating to eight topics: i) farming conditions (location, farm layout, and housing), ii) use of feed, iii) management of water, iv) overall farm management, v) animal health, vi) animal welfare, vii) the environment (in relation to proper disposal of refuse, manure, discarded carcasses, and water treatment) and viii) the keeping of records allowing tracing of animals. The standards outlined in the document are also used as guidelines for responsible agencies such as the Provincial and Regional DLD Livestock Offices to accredit and monitor pig farms [25]. However, in order to assess the epidemiological and environmental risk associated with pig farming, as well as to guide future planning, a thorough understanding of how different pig production systems are geographically distributed is needed.

Over the last few years, the DLD has been undertaking regular, detailed livestock censuses throughout Thailand, thanks to a very large network of volunteers coordinated by regional, provincial, and district veterinary officers. This study aimed to analyze these very detailed census data on pig distributions in Thailand with two objectives. First, we aimed to describe the geographical patterns and trends in pig farming in Thailand in terms of pig breeds, farming systems, and farm scales. Second, we aimed to analyse the spatial distribution of these different systems in relation to spatial factors that may influence their distribution.

## Methods

### Pig and human population data

**Fig. 1:**
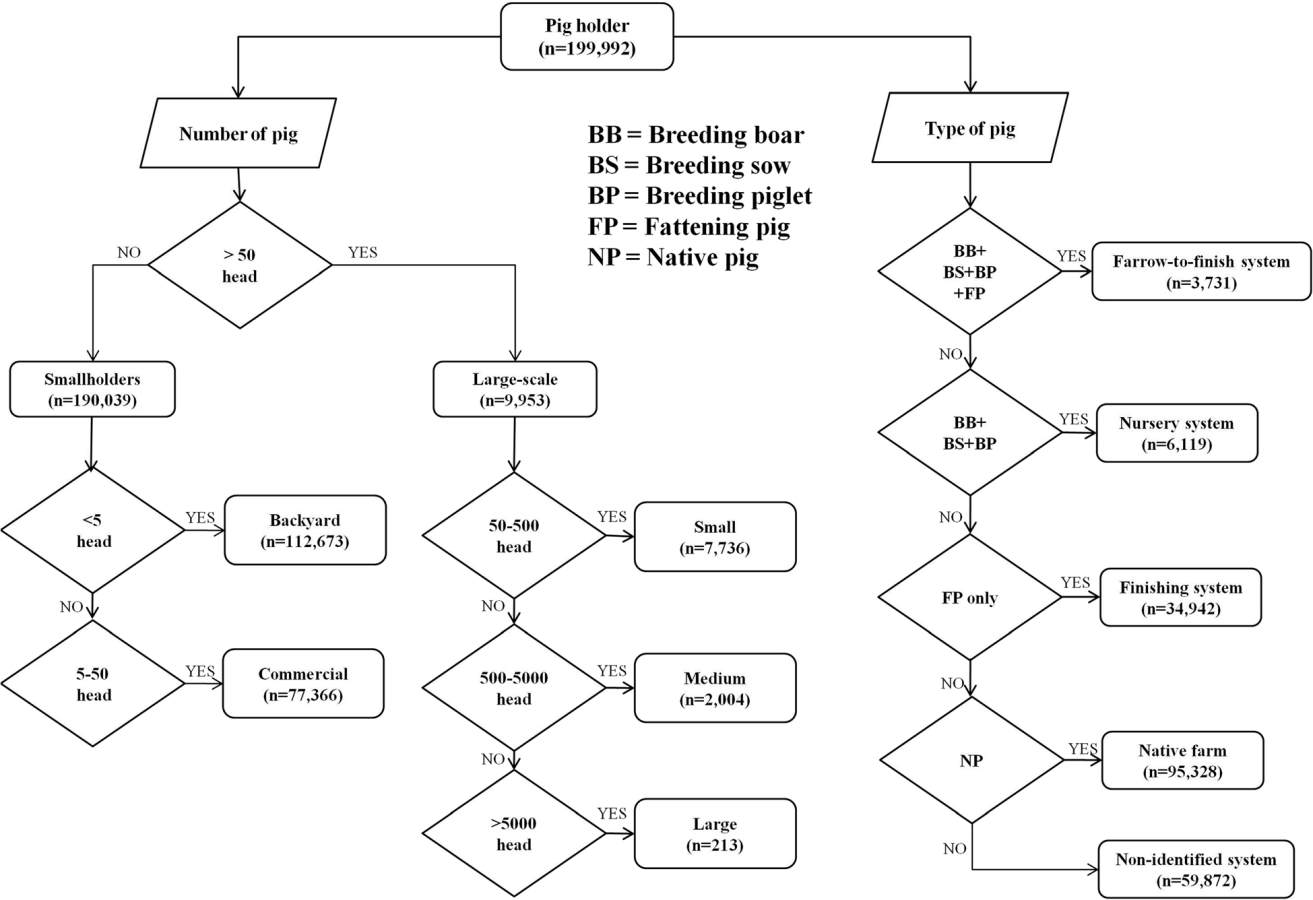
Decision rules identifying pig farming systems. *Left side* shows the proposed classification of the smallholders and large-scale farming systems according to the pig numbers, with holders raising less than 50 pigs being considered as smallholders (<5 pigs per holder for backyard and 5–50 pigs per holder for smallholder commercial) and holders with 50or more pigs considered as large-scale farming system (50–500 pigs per holder for small, 500–5000 pigs per holder for moderate, and >5000 pigs per holder for large). *Right side* shows a proposed classification of farming system according to pig types, with i) farrow-to-finish system if the holder includes all types of breeding pig (boar, sow, and piglet) as well as fattening pigs, ii) nursery system, if the holder includes all types of breeding pig (but no fatting pigs), and ii) finishing system if holder includes only fattening pigs

Throughout the paper, we use the term of “farm” or “holder” to refer to a household keeping at least one pig. Pig population data, both globally and for Thailand during 1964–2013 were obtained from FAOSTAT [26]. More detailed time-series data between 2004 and 2013 on the number of pigs per holder were obtained from the DLD annual census data conducted every year in January [27]. Local DLD staff and livestock volunteers conducted house-to-house census surveys and reported data through a web-based reporting system [27]. The census includes locations (owner name and address), annual counts of native pigs, breeding pigs (boars, sows and piglets), and fattening pigs per holder. The census includes annual counts of native pigs, breeding pigs (boars, sows and piglets), and fattening pigs per holder. There was no strict definition of farming systems used by the pig census so holders were allocated to different farming system according to the following rules, illustrated schematically in Fig. 1. We considered a holder to be of the farrow-to-finish system if its records showed that it was keeping all types of breeding pig (boar, sow, and piglet) as well as fattening pigs. A nursery farming system was assumed for holders keeping all types of breeding pig (but no fattening pigs), whereas a finishing system was assumed for holders keeping only fattening pigs.

Smallholders and large-scale farming systems were separated based on the number of pigs per holder, with holders raising less than 50 pigs being considered as smallholders (<5 pigs per holder for backyard and 5–50 pigs per holder for commercial) and holders with fifty or more pigs being considered as large-scale farming system (50–500 pigs per holder for small, 500–5000 pigs per holder for moderate, and >5000 pigs per holder for large) (the categories shown in Table 1). We previously indicated that farm size is strongly linked to extensive and intensive system. Here, we use the number of 50 pigs per holder with <50 to match the definition of the agricultural standard on the “Good Agricultural Practices for Pig Farms” [25], that is used for operational and management purposes in Thailand. Data on human population counts were provided by the Bureau of Registration Administration (BORA), Department of Provincial Administration [28].

**Table 1:** Criteria to discriminate pig farming systems. Criteria to discriminate pig farming systems using farm scales as defined in the Standard for Good Agricultural Practices for Pig Farm in Thailand in 2009

Categories	Definitions	Approximate number of pigs
Smallholder	Raising boar and sow or finishing pig or piglet or combination of different ages that has the livestock weight less than six units^a^	<50 head^b^
Large-scale farm		
Small	Raising boar and sow or finishing pig or piglet or combination of different phases of age that has the livestock weight between 6 and 60 units^a^	50–500 head
Medium	Raising boar and sow or finishing pig or piglet or combination of different phases of age that has the livestock weight between 60 and 600 units.	500–5000 head
Large	Raising boar and sow or finishing pig or piglet or combination of different phases of age that has the livestock weight more than 600 units	>5000 head

^a^Unit of livestock weight means net weight of boar and sow or finishing pig or piglet or combination of different ages that have total weight equal to 500 kg by assigning 170 kg for the average weight of boar or sow, 60 kg for finishing pig and 12 kg for piglet

^b^50 head calculated from (6 units × 500 kg)/60 kg

### Analysis

Previous studies relating livestock distributions to spatial variables have mostly employed linear regression models [29–32]. For example the global livestock distribution maps provided by the Gridded Livestock of the World 1 (GLW1) [31] and GLW2 [30] were carried out through the use of stratified linear regression models. A similar method was employed to predict the distribution of chickens, ducks and geese in China [29] and to predict the distribution of domestic ducks in Monsoon Asia [32]. A slightly different methodological approach was used to map the distribution of intensive poultry farming in Thailand, through the use of a simultaneous autoregressive model (SAR) that incorporates an explicit component to account for spatial autocorrelation in the linear regression modeling framework [33]. Two different approaches were used to downscale livestock distribution data in Europe: i) an expert-based suitability rule and ii) a statistical modeling approach based on multiple regression [34].

In this study, we used a Random Forest (RF) approach to quantify the association between the predictor variables and the pig population data in 2010. RF is a machine learning method, which combines the prediction of a high number of classification trees in an ensemble, non-parametric approach [35]. The RF algorithm for regression works by: i) drawing *n* bootstrap sub-samples from the original data; ii) growing un-pruned regression trees by randomly sampling *m* variables from a list of predictor variables and choosing the best split from those predictor variables for each of the bootstrap samples (i.e. each tree) and iii) generating a final predicted value by averaging the predictions of the *n* trees [36]. RF estimates the error rate based on the training data that are randomly sampled 36 % of the whole part at each bootstrap iteration (called as “out-of-bag”, or OOB) [35, 36]. The error rate is calculated from the predictions aggregated from all bootstrapped training sets (called as the OOB estimate of error rate). The variable importance is reported by counting the number of time each variable is selected in the different trees, so it is an absolute measure where variables importance is assessed according to their relative contribution [36]. In general, the variable importance may vary from run to run, but the ranking of the variable is generally stable, so these estimates should not necessarily be interpreted in absolute terms. Compared with other methods, RF has a high ability to model complex interactions among predictor variables [37] and was recently shown to provide highly accurate results in modeling livestock [38] and human population [39, 40].

**Fig. 2:**
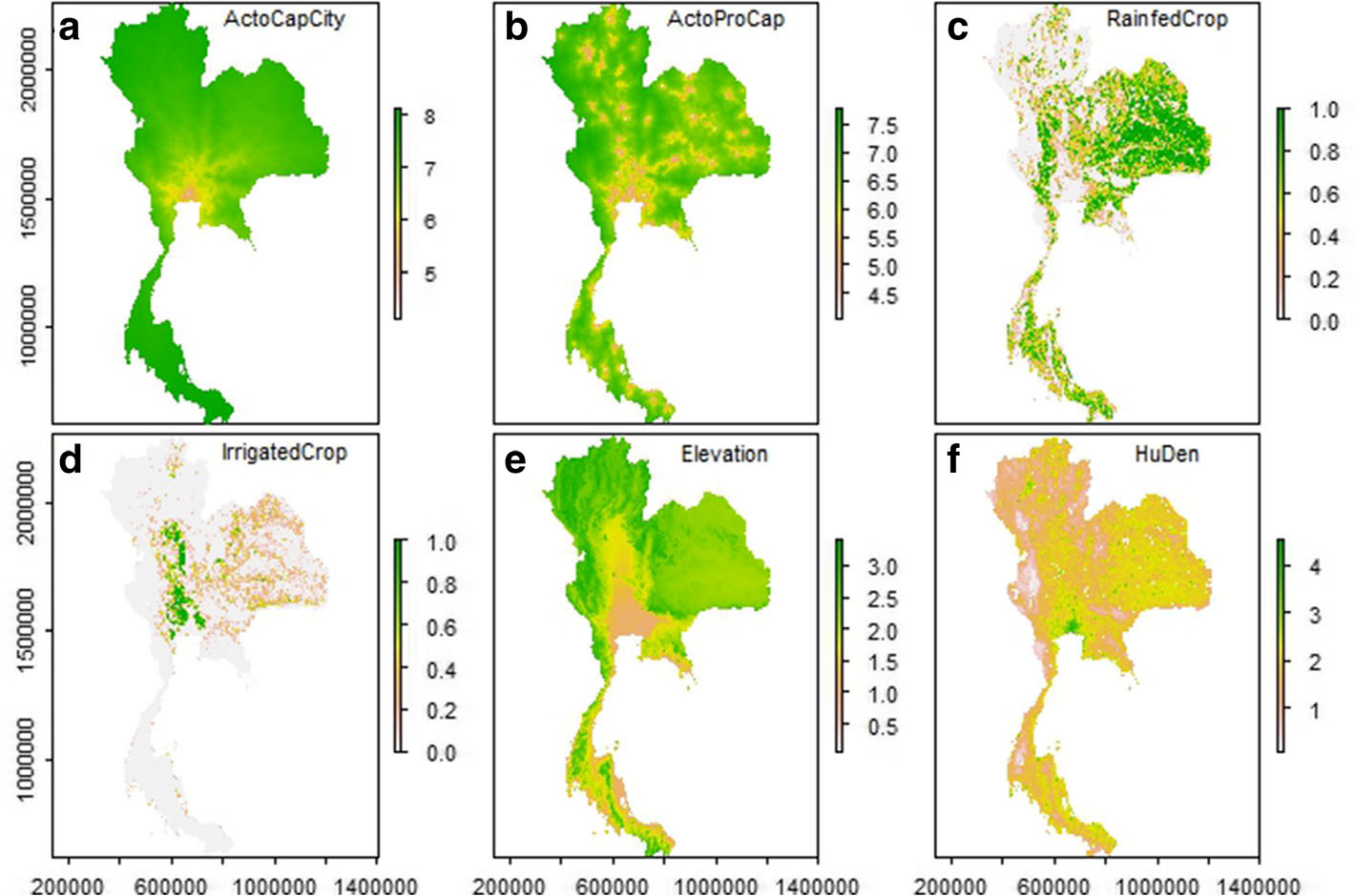
Spatial datasets used as predictor variables for modeling the pig distribution in Thailand. The variables (1 km resolution) include; **a** Travel time to the capital city (Bangkok) (log10 of time) [44], **b** Travel time to the provincial capitals (log10 of time) [44], **c** rainfed croplands (proportion within a square kilometer) [43], **d** irrigated croplands (proportion within a square kilometer) [43], **e** elevation (log10 of meter) [42], and **f** human population density (log10 of number of human per a square kilometer) [39]

Predictor variables used to explain the distribution of pig types and pig farming systems were according to the literature, with variables that may account for market and consumer access (travel time, human population density), local provision of feed (crop) and topographic constrains [2, 33, 41]. Six spatial covariates were included in the model in order to quantify their association with the spatial distribution of different pig production systems (Fig. 2). The covariates were: i) two variables accounting for the spatial distribution of croplands used for animal feed (the proportion of rain-fed croplands within a square kilometre and the proportion of irrigated croplands within a square kilometre); ii) two variables that account for access to urban markets; travel time to provincial capitals and to the capital city of Thailand, Bangkok (iii) human population density; and iv) elevation (to account for the observation that native breeds are usually raised on highland and commercial breeds in the plains). To ensure that predictor variables could generate results potentially comparable with other regions, these were obtained from global or regional datasets. A human population density raster map at 100 m resolution was obtained from the Worldpop project [39]. We used the SRTM elevation database with 90 m spatial resolution produced by NASA [42]. The two maps of croplands at 300 m resolution were extracted from the land cover map obtained from the GlobCover project [43], and each cropland class quantification was computed using a focal mean within 1 km. Travel time (accessibility) was estimated using a travel “friction surface” [44], which was initially created by calculating the total time needed to cross the cell of a raster grid based on ancillary data such as land cover, road type, water bodies and slope. The travelled-time maps were created from the friction surface using a cost-distance algorithm to determine the cost of travelling from each pixel to the closest point of interest; either the province capital or Bangkok. Finally, all raster maps of predictor variables were aggregated to 1 km resolution, and then averaged to sub-district unit. The data processing was implemented in ArcGIS 10.2.

The predictor variables were used to build five separate RF models with the following dependent variables i) native pig density, ii) breeding pig density, iii) fattening pig density, iv) smallholders density, and iv) large-scale pig farms density, with all densities expressed in head per square km. Exploratory data analysis indicated that there was strong over-dispersion, especially in the farm density variables, as well as zero inflation [45].

We dealt with the zero inflation through a zero-altered model (also called a hurdle model or a two-part model) [46–49] where presence/absence was modelled separately from abundance, upon presence. First, a binomial RF model was first constructed to predict zero and non-zeros observations [48, 49]. Indeed, a zero value in the census may occur for a variety of reasons: i) the absence of pig because of unsuitable conditions for farming, such as in the urban areas (structural unsuitability); ii) the conditions were suitable for pig farming but pig was absent at the specific time of the census (e.g. moved out to be slaughtered, design error); iii) a pig was present but the observer misidentified it or missed its presence (observer error); iv) conditions were suitable for pig farming but no farmer had taken it up (farmer error). The zeros due to design, observer and farmer errors are also called false zeros or false negatives and the structural unsuitability are called positive zeros, true zeros, or true negatives [45]. The keystone of the two-part model developed here is that the model does not discriminate between the four different types of zeros. In previous studies having to deal with zero-inflated data, a comparison of five regression models was studied (Poisson, negative binomial, quasi-Poisson, the zero-altered (a two-part model) and the zero-inflated Poisson), the results showed that the zero-altered model performed best, with the highest correlations between the observed and predicted abundances [47]. Second, the non-zero observations were predicted using a quantitative RF model, where we dealt with over-dispersion through a log10 transform (log10(x + 1)). All RF models were built from 500 trees, each bootstrap being predicted by four predictor variables randomly selected from the set of six. The RF models were used to derive predicted density maps both at the sub-district level (using predictors aggregated at the sub-district level) and at the 1 km pixel level (applying the RF model to the 1 km resolution predictors) and the predicted values of each map were then combined. We summed the 1 km cell values within each of the sub-district units.

Two statistical metrics were used to quantify the goodness of fit between observed and predicted densities: the correlation coefficient (COR) and the root mean square error (RMSE). A correlation coefficient provides an indication of precision, i.e. how closely the observed and predicted values agree in relative terms, with a perfect correlation equal to one [47]. RMSE depends on the sample size ($n$), and the discrepancy between the observed (${y}_i$) and predicted ($\widehat{y_i}$) values [47]. It provides an estimate of accuracy and is calculated as:

$$ \mathrm{RMSE}=\sqrt{\frac{1}{n}{\displaystyle {\sum}_{i=1}^n{\left(\widehat{y}-y{}_i\right)}^2}} $$

Analyses were carried out using the “randomForest” [50] and “hydroGOF” [51] packages in R for the RF model and goodness of fit estimates, respectively.

## Results

### The development of pig population in Thailand

**Fig. 3:**
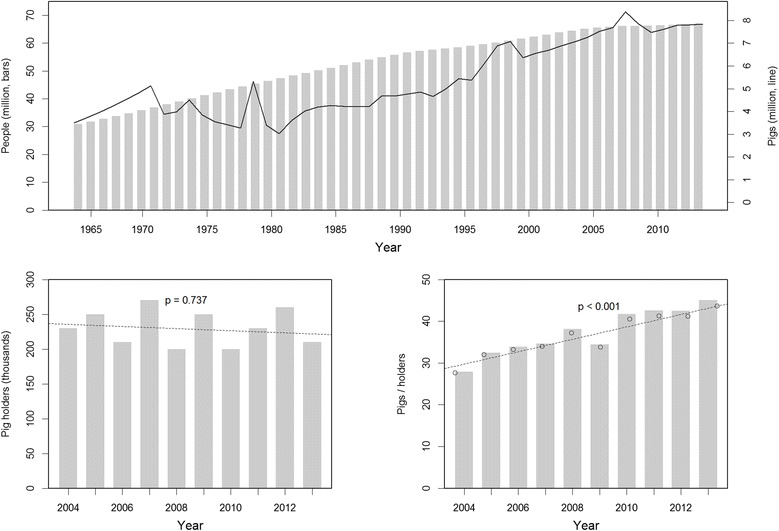
Temporal distribution pattern of pig population. Top shows comparisons between human and pig populations in Thailand over the past 50 years (1964–2013), which bar plot shows the number of human population (*left y-axis*) and line plot shows the number of pig population (*right y-axis*). Left bottom shows trends in numbers of pig holders in Thailand over the past 10 years. Right bottom shows an average size of pig holding in Thailand over the past 10 years

The overall trend in pig production in Thailand over the past 50 years differed from the global pattern. While the global pig population has increased regularly over the past half century, the pig population in Thailand has shown a much more variable trajectory, within an overall trend of increase since the mid-1980s (Fig. 3-Top). The number of pig holders in Thailand remained fairly stable for the last 10 years 2004–2013, but showed an interesting fluctuating pattern (Fig. 3-Left bottom). Intensification of the pig sector can be quantified through the number of pigs per holder, which increased steadily during the same period (Fig. 3-Right bottom). Changes in human and pig population between 2004 and 2013, at the global level and in Thailand, are presented in Table 2. These figures show that while the total Thai population increased, the number of pig holders slightly decreased. The Thai pig population represented 0.70 % of the global pig population in 2004 against 0.97 % of global pig population in 2013. The compound annual growth rate over that period was 4.7 %, with fattening pigs growing by 5.1 % per year, breeding pigs by 4.6 % per year and native pigs by 1.6 % per year. In contrast, the compound annual growth rate of boars decreased by 0.93 % per year. The growth rate of pig holders in Thailand was also negative, with decreases of 0.74, 1.1 and 0.60 % per year for breeding pigs and native pig holders, respectively. Meanwhile, the number of holders of fattening pigs slightly increased by 0.09 % per year.

**Table 2:** Trends of global and Thai pig production during 10 years. Changes in human population and pig population globally and in Thailand between 2004 and 2013

Type	2004		2013		Compound annual growth rate^a^ (%)	
Human population	Person	Household	Person	Household	Person	Household
Global level (million)	6436		7162		1.19	
Thailand (million)	62	18	65	23	0.53	2.76
Pig population	Head	Holder	Head	Holder	Head	Holder
Total global level (million)	873		977		1.26	
Total Thai pigs	6,285,603	225,592	9,511,389	210,978	4.71	−0.74
Native pigs	504,075	86,622	580,069	82,083	1.57	−0.60
Breeding pigs	2,032,561	96,024	3,054,758	87,121	4.63	−1.08
Boars	137,226		126,208		−0.93	
Sows	721,341		885,928		2.31	
Piglets	1,173,994		2,042,622		6.35	
Fattening pigs	3,748,967	79,173	5,876,562	79,843	5.12	0.09

^a^Compound annual growth rate (CAGR) is a business and investing specific term for the geometric progression ratio that provides a constant rate of return over the time period

Detailed data on pig populations by pig types, farming systems and farm scales for 2010 are presented in Table 3. There were 8.3 million head of pigs throughout the country with 5.2 million fattening pigs (62 %), 2.5 million breeding pigs (30 %) and 0.68 million native pigs (8 %). The median number of pigs per holder was five, but when broken down by pig type it was three pigs per holder for native pigs, four pigs per holder for breeding pigs, and eight pigs per holder for fattening pigs. The breakdown of commercial farms was 78 % belonging to the finishing systems, 14 % to nursery systems and 8 % to the farrow-to-finish systems. However, the number of pigs per holder of the farrow-to-finish systems (556) was much higher than that of the nursery systems and of the finishing systems with 96, and 88 pigs per holder, respectively.

**Table 3:** Pig production in Thailand in 2010. Pig production in Thailand in 2010 categorized by pig types, pig farming systems and pig farm scales

Groups	Sub-groups	Total number		Scales									
				<5		5–50		50–500		500–5000		>5000	
		Head	Farm	Head	Farm	Head	Farm	Head	Farm	Head	Farm	Head	Farm
Pig types	All pigs	8,346,614	199,992	285,932	112,673	1,214,288	77,366	1,250,106	7736	2,412,105	2004	3,184,183	213
	Native pigs	681,463	95,328	172,988	68,540	342,745	26,122	63,966	626	47,133	35	54,631	5
	Breeding pigs	2,517,651	83,502	108,957	48,443	490,224	31,533	421,327	2965	670,792	489	826,351	72
	Fattening pigs	5,147,500	56,884	66,397	23,027	468,176	28,610	758,085	3491	1,941,515	1629	1,913,327	127
Farming systems*	Farrow-to-finish	2,074,423	3731	145	31	45,932	1613	229,119	1654	526,654	343	1,272,573	90
	Nursery	590,136	6119	2969	708	77,941	4564	104,334	682	173,181	146	231,711	19
	Finishing	3,063,122	34,942	49,481	17,555	217,052	14,020	548,364	2009	1,381,166	1,302	867,059	56

*Farming systems (farrow-to-finish, nursery, and finishing systems) based on commercial pig breeds only

In terms of farm scales, pig holders were classified as smallholders (95.02 %) and large-scale farming systems (4.98 %). Smallholders can be classified into two groups: backyards (representing 60 % of smallholders) and commercial smallholders (40 % of smallholders). 60.83 % of the backyard holders held native pigs, whereas 42.99 and 20.43 % of holders held breeding and fattening pigs, respectively (the percentages do not sum to 100 % because one backyard holder may have pigs of different types). In contrast, these proportions were 33.76, 40.75 and 36.98 % for the commercial smallholders. Even though there were only 5 % of large-scale farming systems (50 to > 5000), they held 82 % of the total pig stock. Within the 5 % of farms classified as large-scale farming systems, 3.9 % were small (50–500 heads), 1.0 % were moderate (500–5000 heads), and 0.10 % were large (>5000 heads).

### Spatial distribution

**Fig. 4:**
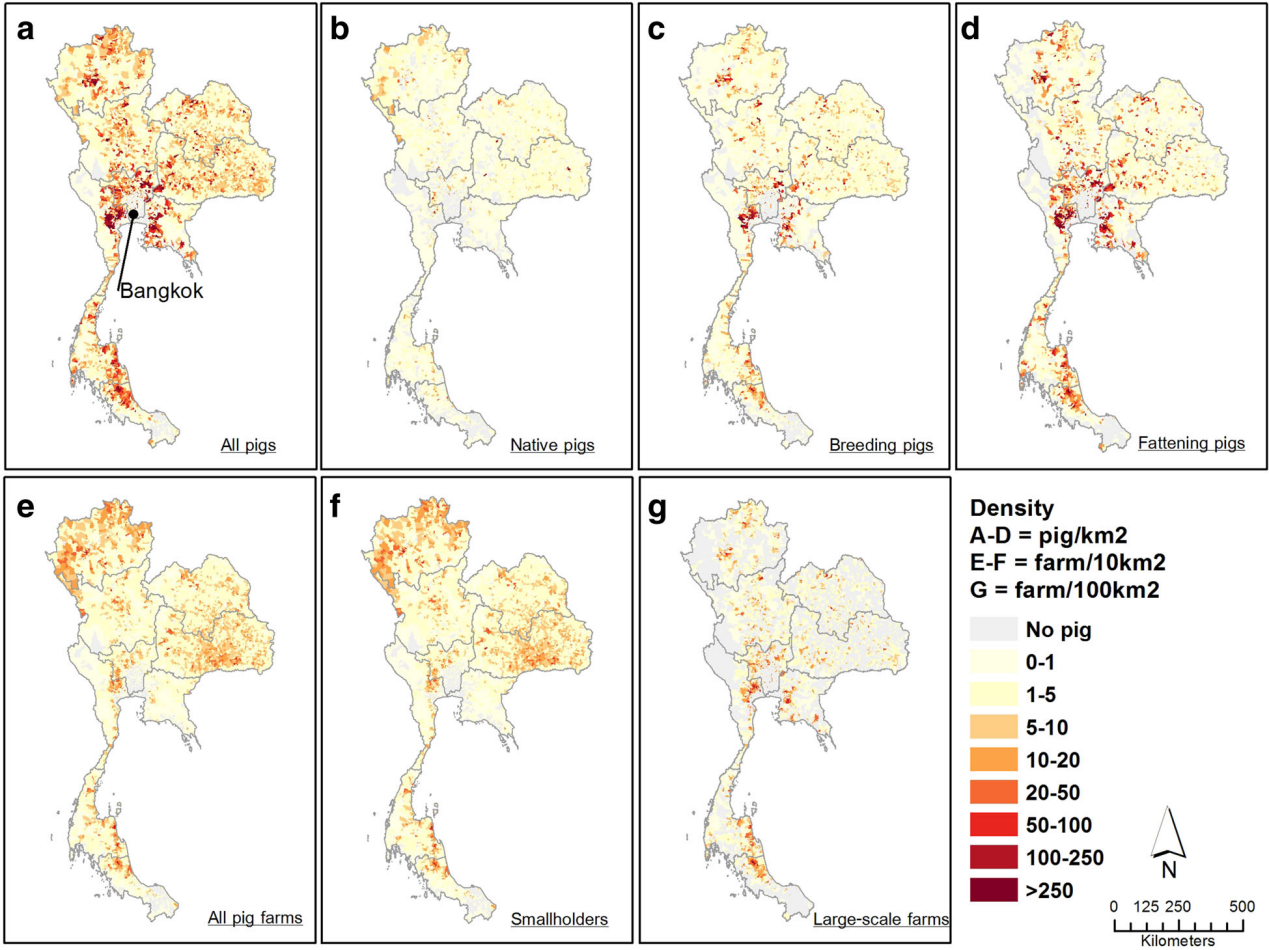
Spatial distributions of pig population in Thailand in 2010. The upper row shows the distribution of pig density by types: all pigs (**a**), native pigs (**b**), breeding pigs (**c**), and fattening pigs (**d**). The lower row shows the distribution of all pig farms (**e**), smallholder farms (**f**) and large-scale farms (**g**) The lower right hand map (**h**) shows the nine regional administrative areas

The spatial distributions of pig population in 2010 were mapped by pig type and farm size (Fig. 4). With a total of 8.3 million pigs in Thailand (Fig. 4a), the highest densities, regardless of pig type, were located in area surrounding the Bangkok Metropolitan region. The lowest densities were found within the city of Bangkok itself, in the three provinces in the lowest areas provinces of Yala, Pattani, and Narathiwat, and the western areas adjacent to Myanmar.

When considering different pig types, native pigs (Fig. 4b) were mostly found in isolated and rural areas of the Northwest (high mountains) and in the Northeast, where plateaus and arid lands dominate the landscape. In contrast, breeding pigs and fattening pigs showed very similar patterns (Figs. 4c and d), with concentrations of high densities in areas surrounding the Bangkok Metropolitan Region, two provinces in the North (Chiang Mai and Chiang Rai provinces), and three provinces at the border between Livestock Region 8 and Livestock Region 9 (Nakorn Sithammarat, Pattalung, and Songkhla provinces).

When broken down by farms size (Fig. 4e and g), smallholders appeared to be relatively homogeneously distributed throughout the country, but with lower densities in the Bangkok Metropolitan Region, the eastern region (Livestock Region 2), the western region (forested areas), and the three provinces in the south. In contrast, intensive larger farms were mostly located in the areas nearby the main cities including the areas surrounding the Bangkok Metropolitan Region, the areas nearby Chiang Mai and Chiang Rai provinces (in the North), and the areas nearby Song Khla province (in the South).

### Distribution modeling

The distribution of absences for different categories was fairly similar for different categories (Additional file 1: Figure S1) with a small minority of sub-districts with no pigs, located either in very remote and inhabited areas, or in very dense urban areas. So, we only report the results of the quantitative part of the zero-altered model, and the equivalent results for the binomial presence/absence models are presented as supplementary information. The importance of different spatial covariates in the quantitative RF models is shown in Table 4 ((Additional file 1: Table S1 for the binomial model). The strongest predictors of distribution for the different pig types and farm scales was the human population density (median variable importance of 66.9 %), followed by travel time to the capital city (median variable importance of 58.7 %), elevation (median variable importance of 44.8 %), travel time to the provincial capitals (median variable importance of 37.6 %), rainfed croplands (median variable importance of 36.8 %), and irrigated croplands (median variable importance of 30.5 %). We obtained better accuracies (Table 4), when predictions were made directly at the sub-district level (with aggregated predictors) rather than by aggregating the results of the pixel-level predictions.

**Table 4:** Important variables modeled by the quantitative Random forests and evaluation of predicted maps modeled by combined models. The variable importance (%) used to predict pig types and pig farm scales and the evaluation of the combined models. Predictor variables include, travel time to the capital city (Bangkok), travel time to the provincial capitals (Meung districts), rainfed croplands irrigated croplands, elevation, and human density)

Categories	Response variables^a^	The variable importance^b^						Evaluation			
		TCapCity	TProCap	RaCrop	IrCrop	Elev	HuDen	RMSE^c^	Correlation	RMSE	Correlation
								(sub-district)	(sub-district)	(pixel)	(pixel)
Pig types (heads/km2)	Native pigs	41.97	27.54	29.44	23.86	34.64	66.89	0.12	0.94	1.19	0.78
	Breeding pigs	58.74	38.84	36.76	30.46	44.82	75.38	0.23	0.91	1.32	0.79
	Fattening pigs	61.16	37.57	47.71	33.07	63.81	63.55	0.31	0.87	1.28	0.83
Pig farm scales (farms/10 km2)	SM	100.27	46.86	62.10	50.90	77.83	148.82	0.14	0.95	1.43	0.80
	LF	21.85	21.68	25.33	15.39	36.33	62.09	0.07	0.92	0.74	0.74

^a^Response variables include: number of native pigs, number of breeding pigs, number of fattening pig, number of smallholders (SM), and number of large-scale farming systems (LF)

^b^Predictor variables include: travel time to the capital city (TCapCity), travel time to the provincial capitals (TProCap), rainfed croplands (RaCrop), irrigated croplands (IrCrop), elevation (Elev), and human density (HuDen)

^c^RMSE stands for root mean square error

**Fig. 5:**
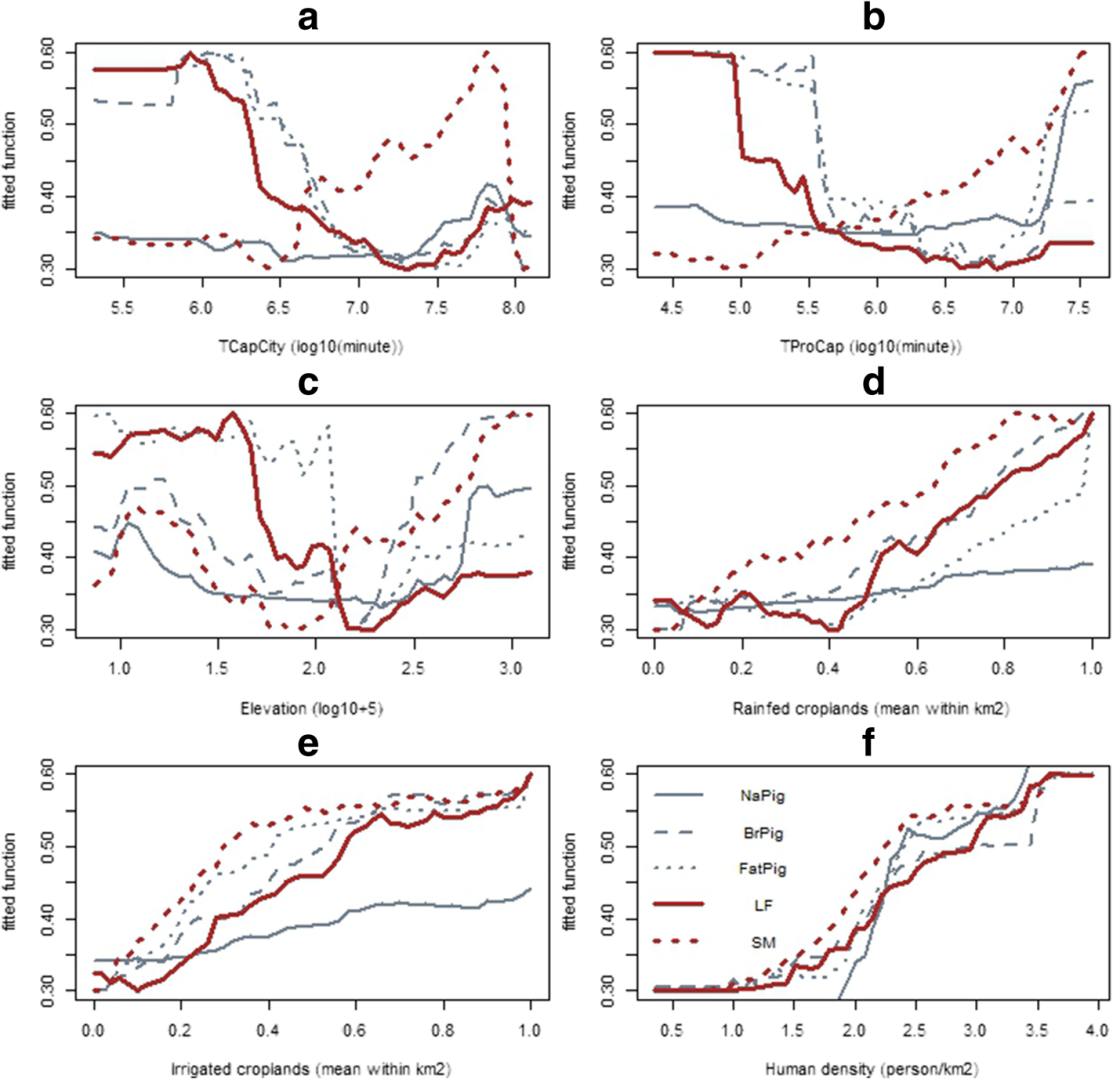
Partial dependent plots of the fitted function (Y-axis) and the predictor variables (X-axis). Response variables include: native pig density (NaPig), breeding pig density (BrPig), fattening pig density (FatPig), Large-scale farm density (LF), and smallholder density (SM). The predictor variables include: **a** travel time to the capital city (TCapCity), **b** travel time to the provincial capitals (TProCap), **c** elevation, **d** rainfed croplands, **d** irrigated croplands, and **e** human population density

The association between the fitted functions and the predictor variables modelled by the quantitative RF model are shown in Fig. 5. The plots show that three variables, including rainfed croplands, irrigated croplands, and human population density shown a similar positive association with the predicted values for all pig farming types (Fig. 5d to f). In contrast, for two predictor variables, the travel time to the capital city and the travel time to the provincial capitals, different relationships were found according to the type of pig farming (Fig. 5a to b). Breeding pigs and fattening pigs showed a negative association with those predictors, whereas native pigs showed an inverse positive association. The same contrasting pattern with these predictors was found for the farm scale categories, where large-scale production systems showed a negative association with travel time to the capital city and travel time to the provincial capitals, whereas smallholders showed a positive association. Regarding elevation (Fig. 5c), fattening pigs and large-scale production systems showed correlation with low elevation, while smallholders, native pigs, and breeding pigs showed both a negative and positive association. In the binomial presence/absence model, such inverse associations were not apparent ((Additional file 1: Figure S2), as there was much more similarity between the presence/absence distributions of the different categories (e.g. probability of absence was predicted to be positively associated with elevation in all categories).

## Discussion

Pig populations in Thailand over the past 50 years showed an initial decline and then increased from the mid-1980s. Interestingly, although the overal mean number of pig owners has remained fairly stable, it has shown strong fluctuations around the mean. This phenomon has been described as the “pork cycle” or “hog cycle” [41] The “pork cycle” in Thailand has been characterized typically by a 32 month cyle, with 16 months of loss and 16 months of profit [41]. The cycles have been attributed to interactions between economic and animal health factors [41]. A large proportion of the stock is held by smallholders who quickly adapt to changing market-prices on the markets. When the prices are high, many smallholders start producing pork. After the time required bringing them to slaughter weight, the supply increases, but the demand remains the same resulting in a drop in price. Smallholders then start losing money and stop raising pigs. This gradually reduces the amount of pork on the market, and the prices revert to higher levels. These fluctuations are not absorbed by export and imports, because majority of the Thai pork production, especially that of smallholders, serves domestic markets. In addition, outbreaks of pig diseases, such as virulent strains of PRRS [11, 12], can have an influence on pork production and market prices, and contribute to trigger or amplify these fluctuations. Interestingly, although the total number of pig holders did not vary so much in time over this study period, the average number of pigs per holding showed an increasing trend, which confirms that Thailand is still intensifying its pig production, and this will impact over longer time period on the number of smallholders, who will gradually either, quit pork production, or move to large-scale pig farming.

The spatial distribution of large-scale farming systems currently largely corresponded to the distribution of commercial pig types (breeding pigs and fattening pigs), in the suburban areas surrounding the main cities, particularly around the Bangkok Metropolitan region. Both large-scale farming systems and commercial pig breeds showed negative associations with travel time to the capital city and to provincial capitals (Meung districts), and with elevation, and a positive association with human density, rainfed croplands and irrigated croplands. This indicates that most of the intensive pig farms and commercial pig breeds were located in suburban areas in lowlands, conveniently placed to transport produce to consumption markets such as provinces capital and the Bangkok Metropolitan region, and at the same time to access a local supply chain of pig feed ingredients [52]. A similar spatial pattern has been observed in a similar study carried out for poultry [33]. The major pig production provinces were previously within 60–150 km of Bangkok but this catchment has now expanded to 250 km because of the rapid increase in demand for livestock products and improvements in transport [2]. In contrast to the spatial distribution of large-scale farming systems, smallholders, comprising 95 % of all farms types, were distributed in the more rural parts of the country. They showed positive associations with all predictor variables, suggesting that the smallholders were more likely to be located in highland, remote, rural areas to supply the rural and local markets. However, the basic requirement of a supply chain for pig feed, suggested by the positive association with croplands, is also necessary for smallholders, a pattern observed also in poultry [33]. So, smallholders and large-scale farms showed inverse relationships for travel times to cities and elevation, but similar relationships with human population density and crop-related variables. These results makes perfect sense, since smallholders need local consumers (hence a positive association with human population density) and local feed supply (hence the positive association with cropland), and are not affected by difficulties of transport encountered in high elevation areas. Conversely, large-scale farming required similar conditions in terms of consumer and feed, but rely so strongly on large consumption centers that market access quantified through travel time become an inverse relationship, i.e. high numbers of farms for short travel time to the province capital or to Bangkok. One should note that the travelled-time layer used here may not adequately reflect the travel constraints of trucks, as it also included landcover in its design. However, we feel that this probably would have a marginal effect on our results given the scale of our analysis that include a very wide gradient of travel time ranging from Bangkok to the remote mountainous areas. We also used a somewhat arbitrary classification threshold for smallholders, matching operational definitions already used in Thailand, but not necessarily matching home-consumption vs. commercial destination of outputs. However, a backyard vs. commercial divide would probably show similar patterns, i.e. a strong association between backyard producers and remote and rural areas.

All types of pigs were found in smallholders, but the majority of native pig farms were of the smallholders (99.3 %). As shown in the distribution map, native pigs were found throughout the country, but with high-density locations in northern Thailand. The native Thai pigs in the northern highland region are raised by smallholders in the hill tribe communities and are important in relation to local customs and religion, where animals are sacrificed for special celebrations such as New Year and weddings [21, 22]. However, it is noteworthy that 0.7 % of the native pigs were still raised in intensive farms; by 626 small farms, 35 moderately sized and five large farms (>5000 head). This is linked to the increasingly popularity of consumption of wild pigs in restaurants, to which some large-scale producers have responded by increasing their production of native pigs [21].

Most of the pig farming systems in Thailand belonged to the finishing systems (78 %) followed by the nursery systems (14 %) and the farrow-to-finish systems (8 %). The average number of pigs per holder in the farrow-to-finish systems (556) was much higher than that of the nursery systems (96) and the finishing systems (88). The farrow-to-finish systems handle all pig production stages. Consequently, they need to have a high level of specialization and a long experience in using modern technologies to increase productivity [41]. They control the entire production chain by adjusting both the number and quality of pigs raised and fattened [2]. In contrast, owners of the finishing systems need to purchase feeder pigs from external sources, which is a more risky strategy; exposing them to fluctuations in supply, unreliable genetic background, and to poor overall quality and health of the animals [2].

The different geographical patterns of large-scale commercial and smallholder production offer opportunities to their future sustainable developments, since better and more sustainable modes of production could be applied to both modes of production.

Small holders pig farmers could integrate pig farming with a combination of other livestock, crops, vegetables and fruit production as an integrated organic farming [53, 54]. The combination of different vegetable and animal products could also cover the family’s consumption needs, and reduce dependency on the sale of products, thus protecting themselves from price fluctuations [54]. In addition, the combination of different farming activities can facilitate synergistic interactions [53]. Pig waste can be used to produce biogas for the household as well as organic fertilizer for plants [55–57]. In turn, crop products and residues can be used as animal feed. So, rather than the waste from pig production becoming a source of air and water pollution, it can be better treated by i) using simple biofilters such as rice straw, coconut husks, wood shavings, rattan strips and oil palm [58]; ii) decomposing the waste using the Effective Microorganisms (EM) [59]; and iii) biodigestion to produce biogas in simple containers [55]. Better knowledge of basic of bio-security could be encouraged to protect smallholder farms from harmful agents. In economic terms, the demand for organic farming products is growing in Thailand and this may present new market opportunities for smallholders [60]. Farmers could also work together under cooperatives in order to increase their negotiation power with buyers. This could potentially lead to more sustainable agriculture, environmental protection and animal welfare for this sector, which could be favored through incentives in some particular regions of Thailand.

In the commercial sector, the concept of “Area Wide Integration” (AWI) could be applied in some areas in Thailand, and be geographically informed by the results of this study. The concept of AWI [4, 18] for the most commercially oriented farming involves integrating a particular livestock activity with other forms of crop farming in a specific geographic area not used for other types of livestock production and away from urban development. Within such areas, facilities involved in the production cycle, such as feed mills, slaughterhouses and processing plants can be established, which can greatly enhance bio-security by securing the area as a “pig-zone”. Proper practice can be carried out within the area, such as farm management, distribution of cropping land, utilization of manure for biogas production and composting.

The results of this study indicate that intensified pig farms are already mostly located in suburban areas in lowlands, in areas that area already conveniently placed to transport pig products to the main markets, and with good access to pig feed ingredients. However, proper geographical planning, accounting for different aspects such as a health, environmental and economic sustainability, remains to be carried out [61] in order better to refine the definition of potentially suitable regions for long-term, more sustainable large-scale and small-scale pig raising in Thailand.

One of limitations of this quantitative assessment is that the identification of the farming system was made through the farm size and composition at the time of the census. Since farming systems were not defined prior to the census, farms were simply classified according to the number of pigs of different types. So, farms where some particular types of pigs would not have been present or raised at the time of the census could be misclassified. For the intensification level, we used the number of pigs on a farm as a classifier, with a threshold of 50 head, to separate smallholders and large-scale production systems. This may not reflect perfectly the actual level of inputs (i.e. intensification) and level of productivity in those farms. A more comprehensive assessment of inputs and productivity would be difficult to implement in census studies, but could rather be the focus of specific surveys, which may be stratified according to the categories outlined here. However, using this threshold to distinguish between both types of farming systems also has advantages including: i) the bio-security systems, enforced by the regulation on the “Good Agricultural Practice for Pig Farm” for the farms keeping more than 50 pigs [25], are differentiable; and ii) the results of the study can be used to support the strategy of the government directly. However, future study on pig systems in Thailand should consider collecting more detailed data on the pig production systems, such as information on inputs (feed, energy, manpower) outputs (volumes, quality), bio-security and disease prevention practices. These data should not only allow a finer definition of the systems within Thailand, but also facilitate the comparison with data from other countries that could be pursued in the future. We used RF to investigate the relationship between the spatial predictor variables and the pig count, as the method was recently shown to clearly outperform other regression-based techniques in large-scale livestock modeling [38]. However, our primary objective of the RF model was not to optimize the predictive power of our model, but rather to quantify how different spatial factors rank against each other’s in best predicting different categories, and to provide a detailed view of their influence on the fitted values, and we feel that it was helpful in this regard too. For example, the possibility to plot the profiles corresponding to different predictor variables (Fig. 5) allows investigating these with great details, and to show some fairly complex patterns (compare e.g. Fig. 5 A large farms LF versus small farms SM). In comparison, a multiple regression, for example, would provide only coefficients that allows to give the overall direction of the association (positive or negative), but would have more difficulties in handling non-linear relationships, or to account for the multiple interactions between variables, which is one of the strength of machine learning techniques (Random Forest or BRT). A limitation, however, is the lack of formal tests allowing quantifying the significance of a particular variable in terms of hypothesis testing. A formal comparison of different modeling options goes beyond the scope of this paper, and several alternatives such as General Additive Models (GAMs) could have been used as alternatives, but we felt that RF provided a good trade-off between the details of the information it provides and ease of implementation.

## Conclusions

Detailed census data and spatial modeling has enabled the geographical and functional characterization of pig farming systems in Thailand. They highlight a process of intensification of the production, with increasing numbers of pigs per owner over time, large-scale pigs farms concentrated around the capital city to supply its demand, with a tendency of being located increasingly far from the center. Their distribution mostly corresponds to that of breeding and fattening pigs of improved breeds. In contrast, smaller-scale producers are distributed in more rural regions, and more strongly concentrated around local province capitals. These historical developments have not resulted from any specific planning in the past, and have resulted in a present distribution that may not be optimal in terms of environment and health impacts, for example. As the sector is still expanding, future developments may benefit from spatially-informed planning accounting for the specific health, environment and economical implications of the different pig production systems recognizing their specificities. This could be achieved, for example, through the promotion of sustainable intensification of small-scale producers to limit their potential local environmental impact, and by the implementation of AWI for the most intensive production sector in geographically limited parts of the country. Defining these areas geographically could be the scope of follow-up works using multiple-criteria decision analysis tools such as to incorporating environment, heath and economic spatial criteria in the decision-making.

## Additional file

Additional file 1: Supplement information shows the results of a binomial RF model which is used to predict zero and non-zeros observations (pig types and pig farm scales). (DOCX 561 kb)

## Abbreviations

AWI: Area Wide Integration
BORA: Bureau of Registration Administration
BRT: Boosted regression tree
COR: Correlation coefficient
CSF: Classical Swine Fever
DLD: Department of Livestock Development
EM: Effective Microorganisms
FMD: Foot and Mouth Disease
GAMs: General Additive Models
GLW1: Gridded Livestock of the World 1
GLW2: Gridded Livestock of the World 2
MOAC: Ministry of Agriculture and Cooperatives
OOB: Out of bag
PCD: Pollution Control Department
PRRS: Porcine Reproductive and Respiratory Syndrome
RF: Random forest
RMSE: Root mean square error
SAR: Simultaneous autoregressive model
TGE: Transmissible Gastroenteritis
