# Supplementary material for: Spatial analysis and characteristics of pig farming in Thailand
Source: BMC Vet Res. 2016 Oct 6;12:218. doi: 10.1186/s12917-016-0849-7 (PMC5053203; doi:10.1186/s12917-016-0849-7)
Supplement: Additional file 1: — Supplement information shows the results of a binomial RF model which is used to predict zero and non-zeros observations (pig types and pig farm scales). (DOCX 561 kb) [file 12917_2016_849_MOESM1_ESM.docx]

**Supplementary information**

**
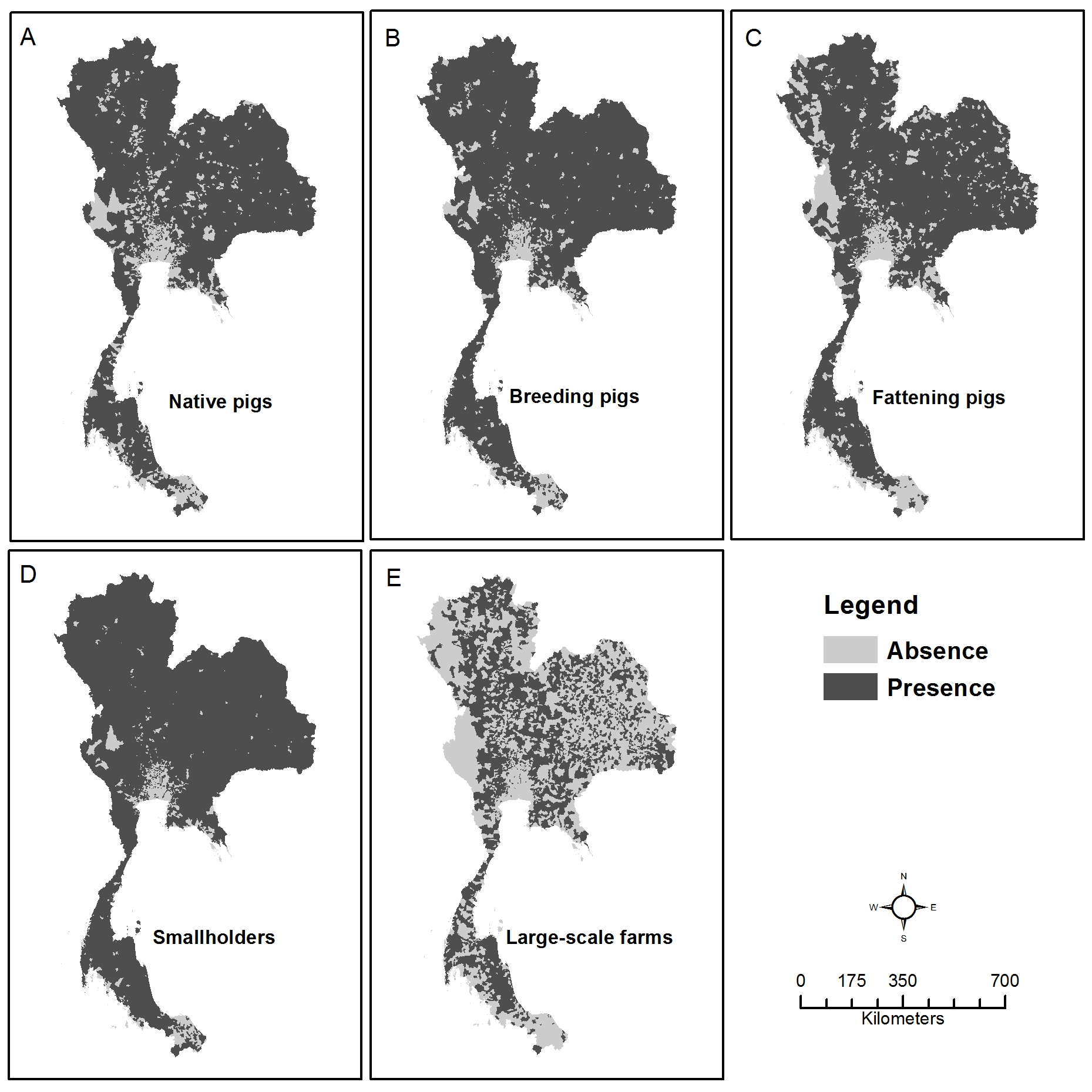
**

**Figure S1. Presence/absence maps of different pig types and pig farm scales.** The maps shows the presences and absences at the sub-district level of pigs belonging to the following categories: native pigs (A), breeding pigs (B), fattening pigs (C), smallholders (D), large-scale farms (E).

**
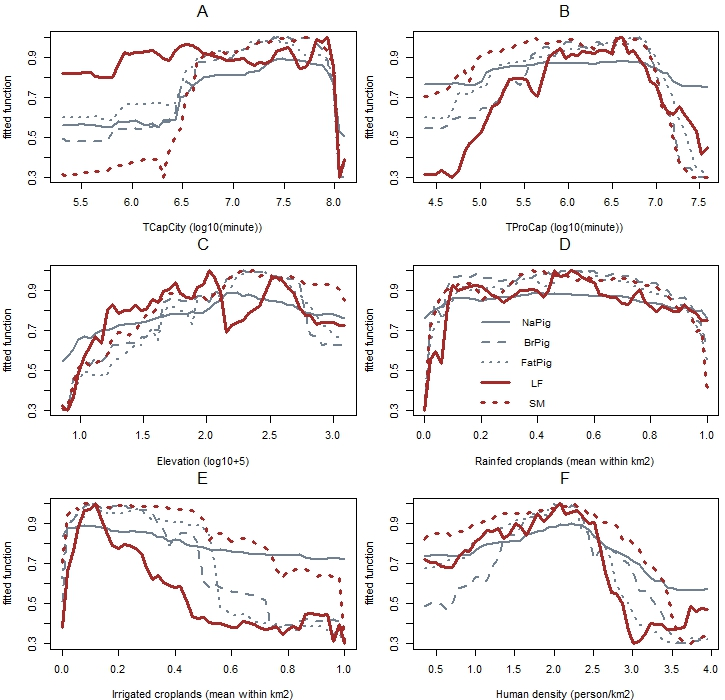
**

**Figure S2 Partial dependent plots of the fitted function (Y-axis) of the absence and the predictor variables (X-axis) of the binomial Random Forest models.** The predictor variables include: native pig density (NaPig), breeding pig density (BrPig), fattening pig density (FatPig), Large-scale farm density (LF), and smallholder density (SM). The predictor variables include: a) travel time to the capital city (TCapCity), b) travel time to the provincial capitals (TProCap), c) elevation, d) rainfed croplands, d) irrigated croplands, and e) human population density.

**Table S1** **Important variables and evaluation of predicted maps modeled by the binomial presence/absence Random Forest models.** IncNodePurity, the fraction of trees in which elements fall in the same terminal node, is used to show the importance of each variable to predict pig types and pig farm scales. Predictor variables include, travel time to the capital city (Bangkok), travel time to the provincial capitals (Meung districts), rainfed croplands irrigated croplands, elevation, and human density). Area under the curve (AUC) of receiver operating characteristic (ROC) and Pearson’s correlation (COR) are the parameters used to evaluate the models.

| Categories | Response variables* | Binomial RF (IncNodePurity) | | | | | | Evaluation | |
| --- | --- | --- | --- | --- | --- | --- | --- | --- | --- |
|  |  | TCapCity | TProCap | RaCrop | IrCrop | Elev | HuDen | AUC  (sub-district) | COR  (sub-district) |
| Pig types (heads/km2) | Native pigs | 342.73 | 165.56 | 165.09 | 113.93 | 319.76 | 213.76 | 0.9999960 | 0.95 |
|  | Breeding pigs | 300.71 | 135.00 | 143.69 | 88.45 | 267.47 | 183.30 | 0.9999858 | 0.95 |
|  | Fattening pigs | 348.66 | 167.95 | 186.94 | 122.38 | 296.71 | 215.53 | 0.9999937 | 0.95 |
| Pig farm scales (farms/10 km2) | Smallholders | 271.85 | 107.54 | 105.85 | 63.01 | 205.52 | 164.21 | 0.9999922 | 0.95 |
|  | Large-scale farms | 329.22 | 253.55 | 271.89 | 200.80 | 279.99 | 272.84 | 0.9999994 | 0.96 |
